# Hydrogen Production in Microbial Electrolysis Cells Based on Bacterial Anodes Encapsulated in a Small Bioreactor Platform

**DOI:** 10.3390/microorganisms10051007

**Published:** 2022-05-11

**Authors:** Irina Amar Dubrovin, Lea Ouaknin Hirsch, Shmuel Rozenfeld, Bharath Gandu, Ofir Menashe, Alex Schechter, Rivka Cahan

**Affiliations:** 1 Department of Chemical Engineering and Biotechnology, Ariel University, Ariel 40700, Israel; irinadubrovin@gmail.com (I.A.D.); lea.ouaknin@gmail.com (L.O.H.); shmulik2009@gmail.com (S.R.); bharathgandu@gmail.com (B.G.); 2 Department of Environmental Studies, University of Delhi, New Delhi 110007, India; 3 Water Industry Engineering Department, Engineering Faculty, Kinneret Academic College on the Sea of Galilee, Galilee 1513200, Israel; ofirmn@mx.kinneret.ac.il or; 4 BioCastle Water Technologies Ltd., Afikim 1514800, Israel; 5 Department of Chemistry, Ariel University, Ariel 40700, Israel; salex@ariel.ac.il

**Keywords:** microbial electrolysis cell, bacterial anode, small bioreactor platform, anode encapsulation

## Abstract

Microbial electrolysis cells (MECs) are an emerging technology capable of harvesting part of the potential chemical energy in organic compounds while producing hydrogen. One of the main obstacles in MECs is the bacterial anode, which usually contains mixed cultures. Non-exoelectrogens can act as a physical barrier by settling on the anode surface and displacing the exoelectrogenic microorganisms. Those non-exoelectrogens can also compete with the exoelectrogenic microorganisms for nutrients and reduce hydrogen production. In addition, the bacterial anode needs to withstand the shear and friction forces existing in domestic wastewater plants. In this study, a bacterial anode was encapsulated by a microfiltration membrane. The novel encapsulation technology is based on a small bioreactor platform (SBP) recently developed for achieving successful bioaugmentation in wastewater treatment plants. The 3D capsule (2.5 cm in length, 0.8 cm in diameter) physically separates the exoelectrogenic biofilm on the carbon cloth anode material from the natural microorganisms in the wastewater, while enabling the diffusion of nutrients through the capsule membrane. MECs based on the SBP anode (MEC-SBPs) and the MECs based on a nonencapsulated anode (MEC control) were fed with *Geobacter* medium supplied with acetate for 32 days, and then with artificial wastewater for another 46 days. The electrochemical activity, chemical oxygen demand (COD), bacterial anode viability and relative distribution on the MEC-SBP anode were compared with the MEC control. When the MECs were fed with artificial wastewater, the MEC-SBP produced (at −0.6 V) 1.70 ± 0.22 A m^−2^, twice that of the MEC control. The hydrogen evolution rates were 0.017 and 0.005 m^3^ m^−3^ day^−1^, respectively. The COD consumption rate for both was about the same at 650 ± 70 mg L^−1^. We assume that developing the encapsulated bacterial anode using the SBP technology will help overcome the problem of contamination by non-exoelectrogenic bacteria, as well as the shear and friction forces in wastewater plants.

## 1. Introduction

Large amounts of electrical energy are spent globally to operate domestic wastewater treatment plants (air blowers, hydraulics, and waste sludge stabilization and dehydration). Using appropriate technologies, the potential chemical energy contained in the organic compounds in domestic wastewater might help to improve the energy yield and cost efficiency of treatment plants [1,2]. Among the several chemicals that may be extracted from wastewater, hydrogen occupies a preeminent position because of its desirable potential as a fuel: it is a clean and neutral energy carrier, and can be converted directly into electrical energy very efficiently using fuel-cell technology [3]. Currently, most hydrogen production occurs via water splitting, and is considered a clean energy source. However, this technology is based on applying high voltage (1.5–2 V) and/or high temperatures (225–475 °C) [4,5].

Bioelectrochemical systems (BESs) in general, and microbial electrolysis cells (MECs) [6,7], as well as microbial fuel cells (MFCs) [8] in particular, represent an emerging technology capable of harvesting part of the potential chemical energy in organic compounds. The anodic reactions in MFCs and MECs are quite similar. Almost any source of organic matter, such as the carbohydrates and lipids usually found in wastewater, can serve as a suitable carbon source for bacterial anode activity [7,9,10]. In both MFCs and MECs, the bacterial anode activity leads to the generation of electrons and protons. The main differences between these technologies are in the cathode activity and the operation mode. In MFCs, the presence of an oxidative agent (oxygen) causes the electrical current to flow spontaneously [11,12], whereas MECs require a certain amount of electrical input (about 0.3–0.8 V) to drive the redox reactions [13,14,15]. Electricity and hydrogen are the outcome of MFCs and MECs, respectively. Therefore, they both have the ability to convert some of the negative-value waste streams into value-added products.

Since a BES as a stand-alone treatment presents limited capabilities in removing carbon and nitrogen, it is likely that a BES operated in a wastewater treatment plant would require an additional treatment step. This leads to the operational concept that a BES application, which consists of bioelectrochemical treatment, can be operated in the anaerobic pond, prior to the anoxic pond, followed by an aerobic step [16]. Moreover, it is essential that MEC and MFC technology designs be suitable for long-term implementation in the continuously hostile environment found in domestic wastewater treatment plants.

One of the main obstacles that has been identified is the bacterial anode of a BES, which usually contains mixed cultures in addition to the exoelectrogenic microorganisms [17]. Non-exoelectrogenic microorganisms can act as a physical barrier by settling on the anode surface and displacing the exoelectrogenic microorganisms. In addition, these non-exoelectrogenic microorganisms can induce negative interactions with the exoelectrogenic culture, such as competition for nutrients and carbon sources. These phenomena can reduce electron transfer from the exoelectrogenic bacteria to the anode material [18]. In addition, there are obstacles related to the operational needs of domestic wastewater plants that can reduce the feasibility of BES implementation. These can include shear forces, friction forces, and unstable inflow rates [19].

The anode material in BESs should be highly conductive and biocompatible. Carbon-based materials are commonly used as anode materials due to their chemical stability, conductivity, and cost-effectiveness [20,21,22,23]. However, the carbon materials’ hydrophobicity prevents stable bacterial attachment. A pretreatment with acid and high temperature was reported to overcome the hydrophobicity patterns of carbon materials. For example, high temperature in a muffle furnace at 450 °C for 30 min was used by Wang et al. (2009) [20]. Soaking in acid (H_2_SO_4_) while heating was used by Scott et al. (2007) [24]. However, these pretreatments are performed in extreme conditions. The environmentally friendly method of employing cold nitrogen plasma showed enhancement of the hydrophilicity of a carbon-felt anode, which led to better biofilm formation [25]. Other ways to increase bacterial attachment include the immobilization of bacteria with organic polymers. Gandu et al. (2020) immobilized *G. sulfurreducens* on a carbon-cloth anode using alginate and chitosan. When the MEC, which was based on the immobilized bacterial anode, was fed with wastewater, the current density at a potential of 0.2 V was 11.52 A m^–2^, 29% higher than the non-immobilized anodes, and the hydrogen evolution rate (HER) was 0.56, m^3^ m^−3^ d^−1^, 70% higher than the non-immobilized anode [26]. Another approach to immobilizing the bacterial anode was suggested by Rozenfeld et al. (2021). In their study, the bacterial anode was encapsulated in a dialysis bag. The anode material was made of carbon cloth combined with stainless steel, encapsulated in a dialysis bag including a suspension of *G. sulfurreducens.* The current densities obtained at 0.6 V vs. Ag/AgCl were 16.34 ± 0.42 A m^−2^, whereas the MEC that employed a nonencapsulated anode led to only 12.19 ± 0.49 A m^−2^ [27].

To protect the exoelectrogenic bacterial anode from the shear and friction forces found in domestic wastewater treatment plants, a novel encapsulation technology in a small bioreactor platform (SBP) was adopted. The SBP technology was recently developed for achieving successful bioaugmentation in wastewater treatment plants. The SBP method is based on macro-encapsulation of bacterial culture in a confined environment using a microfiltration membrane as a protective barrier. A 3D capsule (2.5 cm in length, 0.8 cm in diameter) physically separates the bacterial culture, including the carbon cloth-anode material, from the natural microorganisms in the wastewater, while enabling the diffusion of nutrients through the capsule membrane [19]. The SBP capsule creates an appropriate growth environment by providing nutrients and physical protection and preventing competition with natural microorganisms in wastewater plants. This results in rapid biomass acclimation within the SBP capsules. Furthermore, the physical barrier prevents the washout of the selected microorganisms from the bioreactor in a continuous outflow [28].

Our study adopted SBP technology to operate as an anode for a novel MEC. The MEC based on the SBP anode was fed with *Geobacter* medium supplied with acetate for 32 days, and then with artificial wastewater for another 46 days. The electrochemical activity, COD removal, bacterial anode viability, and relative distribution of the MEC-SBP anode were compared with an MEC based on a nonencapsulated anode.

## 2. Materials and Methods

### 2.1. SBP Anode Preparation

**Figure 1:**
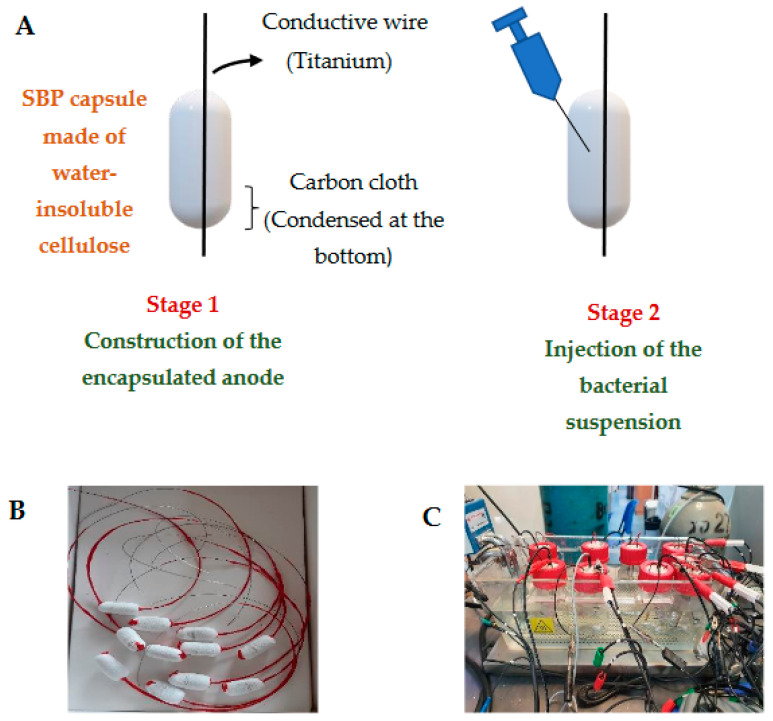
MEC based on the SBP anode. Scheme of the encapsulation anode preparation (**A**); Photo of the anode (**B**); MEC facilities with the encapsulated anode, based on the SBP technology (**C**).

The anode material was made of carbon cloth (E-TEK W1400 LT, Richardson, TX, USA) with a size of 3 × 1 cm (3 cm^2^). A gelatin capsule (size 000, Capsuline, Dania Beach, FL, USA) was used to encapsulate the carbon-cloth anode material, which was then coated with water-insoluble cellulose acetate to construct the membrane (thickness 400–600 µm, pore size 0.2–0.8 mm). The 3D profile of the capsule was 2.5 cm long and 0.8 cm in diameter [19]. The capsule was designated a small bioreactor platform (SBP) as described in patent application PCT IL2010/256 (W.O./2010/122545). After the membrane stabilized, a titanium conductive wire was inserted into and across the SBP capsule and attached to the carbon-cloth anode (designated as the SBP anode, described in patent PCT/IL2021/050911). The protruding portion of the titanium wire was isolated with a synthetic polymer (Plastidip, Yokneam Illit, Israel) to avoid electron diffusion into the medium. The anode was stored in a dry state until inoculation. An illustration and pictures of the SBP anode are presented in Figure 1.

### 2.2. SBP Anode Activation

The capsule, including the carbon-cloth anode, was inserted into distilled water (100 mL) at 50 °C for 5 h (3 repetitions) until most of the gelatin was dissolved. At this stage, the capsule was ready for inoculation with a culture of *G. sulfurreducens.*

### 2.3. Inoculation of G. sulfurreducens into the MEC-SBP Anode and the MEC Control

A pure culture of *G. sulfurreducens* (DSMZ 12127) was grown in *Geobacter* medium (N’ 826, DSMZ, Braunschweig, Germany), under an atmosphere of 80% N_2_: 20% CO_2_, in a 50 mL borosilicate glass serum bottle with a 20 mm butyl septum (Wheaton Glass Co., Millville, NJ, USA) for about ten days, until red bacterial aggregates settled on the bottom of the bottle [29]. The supernatant was decanted, and the highly concentrated bacterial suspension in the bottle was agitated for several minutes. Culture density was measured using a GENESYS 10S UV-Visible spectrophotometer (Thermo Scientific, Waltham, MA, USA) at 590 nm. A volume of 0.6 mL of bacterial culture (1.0 ± 0.05 OD) was injected directly into the MEC-SBP anode with a syringe and needle, creating a pinhole. The membrane pinhole was sealed with cellulose acetate polymer. Meanwhile, the MECs that were based on bare anodes (MEC controls) were inoculated with the same volume and bacterial density into the whole liquid volume (100 mL).

### 2.4. Preparation of Artificial Wastewater

*Staphylococcus aureus* (ATCC, 25923, Manassas, VA, USA), *Escherichia coli* (6899, DSMZ, Germany), *Enterobacter cloacae* (30054, DSMZ, Germany), and *Pseudomonas putida* (6125, DSMZ, Germany) were grown to the log phase in 15 mL Lauria broth (LB) (Himedia, M575, Thane West, India) in 50 mL tubes for about 5 h. Each culture was diluted to 0.8 ± 0.05 OD.

The artificial wastewater included 1 mL of each strain in LB (total of 4 mL) along with 10 mL yeast extract (7184A Neogen, Lexington, KY, USA), 69 mL *Geobacter* medium, and 17 mL phosphate buffer (PB) pH 6.8.

### 2.5. MEC Setup

A single-chamber MEC (100 mL glass bottle) equipped with a septum cap (silicone/PTFE) was filled with 80 mL *Geobacter* medium (N’ 826, DSMZ, Germany) and a phosphate buffer (final concentration of 100 mM, pH 6.8 (20 mL)). It is essential to note that the MEC-SBP and MEC-control components were not sterilized. For the chronoamperometry study, the MEC was connected to a MultiEmStat3+ potentiostat (Palmsens, Houten, The Netherlands) in a three-electrode configuration: a Pt-catalyzed cathode, comprised of carbon cloth coated with 0.5 mg cm^−2^ Pt/60% on carbon support (CTM-GDE-02, FuelCellsEtc, College Station, TX, USA), with a geometric area of 6.25 cm^2^ (2.5 cm × 2.5 cm); an Ag/AgCl 3 M KCl reference electrode (RE-1CP, ALS, Tokyo, Japan); and an SBP anode or control anode (nonencapsulated anode). The MECs were operated under a constant potential of 0.3 V vs. Ag/AgCl (3 M KCl). The distance between the anode and cathode was about 1 cm. In the MECs using the SBP anode, a culture of *G. sulfurreducens* was injected into the SBP-anode, while the MEC controls were inoculated into the whole liquid volume. All MECs were fed with acetate or artificial wastewater.

Five replicates of the MEC-SBP and MEC control were placed in a thermostatic bath at 37 °C. The MECs were connected to a MultiEmStat3+ potentiostat (Palmsens, The Netherlands) in a three-electrode configuration, as described earlier, and were operated under a constant potential of 0.3 V vs. Ag/AgCl (3 M KCl) for 78 days.

### 2.6. Electrochemical Measurement and Chemical Analyses

In the anode electrochemical study experiments, the MECs were connected to a MultiEmStat3+ potentiostat (Palmsens, The Netherlands) in a three-electrode configuration. Linear sweep voltammetry (LSV) was performed in the potential range of −0.5 to 0.8 V vs. Ag/AgCl (3 M KCl) at a scan rate of 5 mV s^−1^ to allow a steady-state polarization [30,31].

Cell electrochemical measurements were performed by connecting the MEC to a MultiEmStat3+ potentiostat (Palmsens, The Netherlands) in a typical two-electrode configuration. A Pt-catalyzed carbon cloth cathode, and an SBP or control anode were measured under steady-state conditions. Slow scan Linear Sweep Voltammetry (LSV) experiments were performed in the applied potential range of 0 to −0.8 V at a scan rate of 5 mV s^−1^ to allow steady-state polarization conditions. Hydrogen production was calculated at given voltages according to Equations (1) and (2), adopted from Logan (2008) [3].

(1) $$V_{H_{2}}=\frac{I\times t\times R\times T}{z\times F\times P}$$

where $V_{H_{2}}$ is the hydrogen production volume (m^3^ s^−1^), *P* is the gas pressure (atm), *V* is the gas volume (m^3^), *z* is the valence of an element, *R* is the gas constant (0.0820577 L atm mol^−1^ K^−1^), *T* is the gas temperature (K), *I* is the current (A), *t* is the time (s), and *F* is the Faraday’s constant (96,485 C mol^−1^).

(2) $$Q{\left(V_{r}\right)}_{H_{2}}=V_{H_{2}}\left(m^{3}\right)\times t\;(d^{-1})\times V_{r}\;\left(m^{-3}\right)$$

where $Q{\left(V_{r}\right)}_{H_{2}}\mathrm{is}\;\mathrm{the}$ HER production rate per cubic meter of the MEC medium, $V_{H_{2}}\;\mathrm{is}\;\mathrm{the}$ hydrogen production volume (m^3^ s^−1^_,_ calculated from Equation (1)), *t* is the time in seconds normalized to 24 h, and $V_{r}\;\mathrm{is}\;\mathrm{the}$ reactor volume normalized to cubic meters (m^3^).

### 2.7. Examination of the Bacterial Anode Biofilm Viability via MTT Assay

At the end of the MECs’ operation, the biofilm viability on the anodes was examined, using the reagent 3-(4,5-Dimethylthiazol-2-yl)-2,5-diphenyltetrazolium bromide (MTT; Merck, Darmstadt, Germany). The MTT assay is based on the bacterial hydrogenase activity, which reduces the tetrazolium reagent. In its oxidized form, the tetrazolium solution is yellow, and in its reduced form, it turns purple. The absorbance intensity of the purple color was measured with a GENESYS 10S UV-Visible spectrophotometer (Thermo Scientific, USA). Each anode (3 cm^2^) with its attached biofilm was washed three times with PBS to remove planktonic bacteria. The anode was transferred to 15 mL tubes containing 5 mL of MTT solution (500 ppm) and was incubated for 2 h at 30 °C in the dark, then the MTT solution was removed and replaced by 5 mL of dimethyl sulfoxide:EtOH solution (1:1 ratio) for 20 min. The absorbance of the purple solution was examined at 540 nm [32,33].

### 2.8. Relative Microbial Distribution Analysis of Bacterial Anode Community

Relative microbial distribution analysis was performed on the SBP and control anodes by HyLabs Ltd., Rehovot, Israel. The DNeasy Powersoil kit (Qiagen, Hilden, Germany) was used to extract the DNA. A 16s library preparation for sequencing on Illumina was performed using a 2-step PCR protocol. In the first PCR, the v4 region of the 16s rRNA gene was amplified using the 16s 515F and 806R from the Earth Microbiome Project, with CS1 and CS2 tails. The second PCR was done using the Fluidigm Access Array primers for Illumina, to add the adaptor and index sequences. Sequencing was done on an Illumina Miseq, using a v2-500-cycles kit to generate 2 × 250 paired-end readings. Demultiplexing was performed on Basespace (the Illumina cloud) to generate FASTQ files for each sample. The data were further analyzed using CLC bio to generate OTU and Abundance tables [26].

### 2.9. Chemical Oxygen Demand Assay

Examination of the chemical oxygen demand (COD), the amount of dissolved oxygen in water needed for oxidizing chemical organic components, was performed using a kit tube (Lovibond™ COD MR, Amesbury, UK). A volume of 2 mL of medium from each MEC was filtered (0.22 μm), diluted and added to the kit tube containing potassium dichromate, sulphuric acid, and metal salts. Potassium dichromate turns from yellow to dull green in its oxidized form, depending on the organic matter concentration. The sample was mixed gently and transferred to a COD reactor (COD reactor DBR-001, MRC, Israel) for 2 h at 150 °C. The digested solution was analyzed using a spectrophotometer (430–610 nm range) (Photometer-system MD 100, Lovibond™, Dortmund, Germany).

### 2.10. Scanning Electron Microscopy (SEM) Analysis

At the end of the MECs’ operation, the bacterial anodes of the MEC-SBPs and MEC controls were gently washed (×3) with PBS. The bacterial anodes were fixed by incubation in Karnovsky’s fixative solution (mixture of 5% glutaraldehyde and 4% formaldehyde in 0.064 M phosphate buffer, pH 7.2), followed by incubation for 1 h in tannic acid (1%) and OsO_4_ (4%) to prevent bacterial cell shrinkage and thermal damage. The samples were washed three times with PBS (pH 7.2) after each process. Finally, they were dehydrated using ethanol (30–100%) and acetone (50–100%) for 10 min at each concentration. The samples were air-dried and sputtered with gold. The morphology of the anodes was examined using a MAIA3 SEM at ultra-high resolution (TESCAN, Brno, Czech Republic) [26].

### 2.11. Statistics

Data were expressed as means ± STDEV function (standard deviation) of between 3–5 replicates. The results were statistically analyzed using a one-way analysis of variance (ANOVA). Differences between the values were considered significant at *p*-value < 0.05.

## 3. Results and Discussion

### 3.1. LSV Measurements of MECs Which Were Fed with Acetate as the Sole Carbon Source

**Figure 2:**
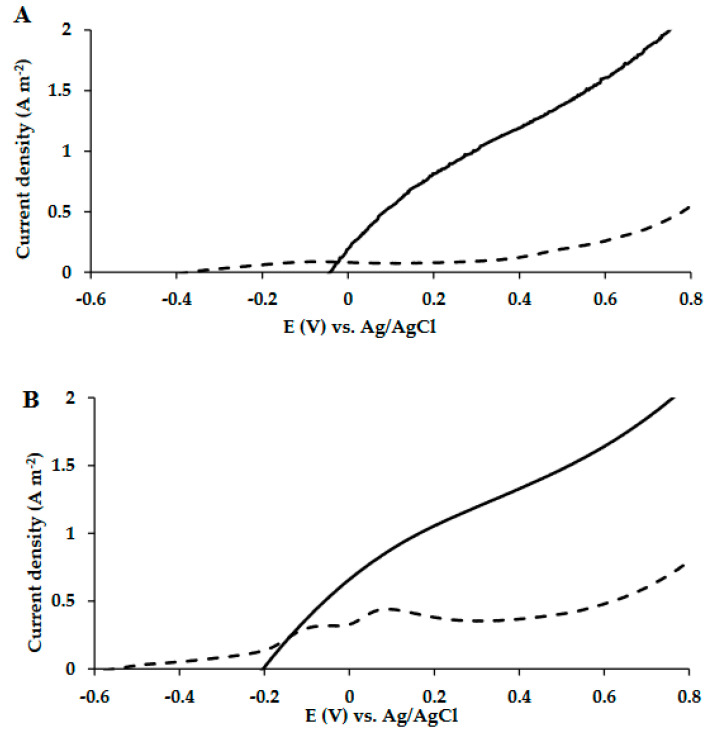
LSV polarization curves of MEC-SBP (**—**) and MEC control (**- - -**) supplied with *Geobacter* medium, and acetate as the sole carbon source. LSV analysis was carried out on the 14th (**A**) and 27th (**B**) days of the MECs’ operation, at a scan rate of 5 mV s^−1^ versus Ag/AgCl.

A single-chamber MEC was constructed based on a carbon-cloth anode encapsulated in an SBP (SBP anode) and a platinum-coated carbon-cloth cathode; designated as MEC-SBP. An MEC with the same cathode and anode materials, but without encapsulation in SBP, served as a control, defined as the MEC control. The MEC-SBPs were inoculated with 0.6 mL (1.0 ± 0.05 OD) of *G. sulfurreducens* culture injected directly into the SBP capsule. Thus, the exoelectrogenic *G. sulfurreducens* were in a confined environment next to the carbon-cloth anode material. The MEC controls were inoculated by injection into the medium of the MEC facility (100 mL). The medium was replaced once a week, and twice a week the MECs were fed with acetate. The MECs were provided with acetate as the sole carbon source for 32 days and maintained under an external voltage of 0.3 V vs. Ag/AgCl. LSV measurements were performed once a week. The LSV analyses on the 14th and 27th days are shown in Figure 2A,B, respectively.

The results depicted in Figure 2A,B show that the MEC-SBP, based on the encapsulated anode, led to higher currents compared to the MEC control. On the 14th and 27th days, under an applied voltage of 0.6 V vs. Ag/AgCl, the current densities of the MEC-SBP were 1.61 ± 0.11 and 1.64 ± 0.29 A m^−2^, respectively. In comparison, the MEC control yielded currents of only 0.25 ± 0.02 and 0.48 ± 0.02 A m^−2^, respectively. The higher observed currents of the SBP anode occurred despite the high onset potential (−0.2 V) compared with the control (−0.55 V); and are in line with the low resistance (ca. 0.4 Ω m^2^), which was 6.25 times higher than the control (2.5 Ω m^2^) (Figure 2B).

The encapsulated anode exhibited electrochemical behavior different from the free-standing carbon electrode, as seen in their onset potentials and the above-mentioned currents. While the exact reason is not clear to us, we think it had to do with the entrapped bacterial cells, their released redox-active mediators, and the interaction of these two with the active biofilm on the carbon electrode inside the capsule. The enrichment of this micro-environment altered the equilibrium potential (and consequently the onset potential), but it also provided a more accessible and higher concentration of bio-electrochemical active species to support a high current. These conditions did not exist in the diluted supernatant surrounding the carbon-cloth anode in the MEC control, which was a free-standing electrode under the same physical and chemical conditions.

### 3.2. LSV Measurements of MECs Utilizing Artificial Wastewater as Carbon Source

**Figure 3:**
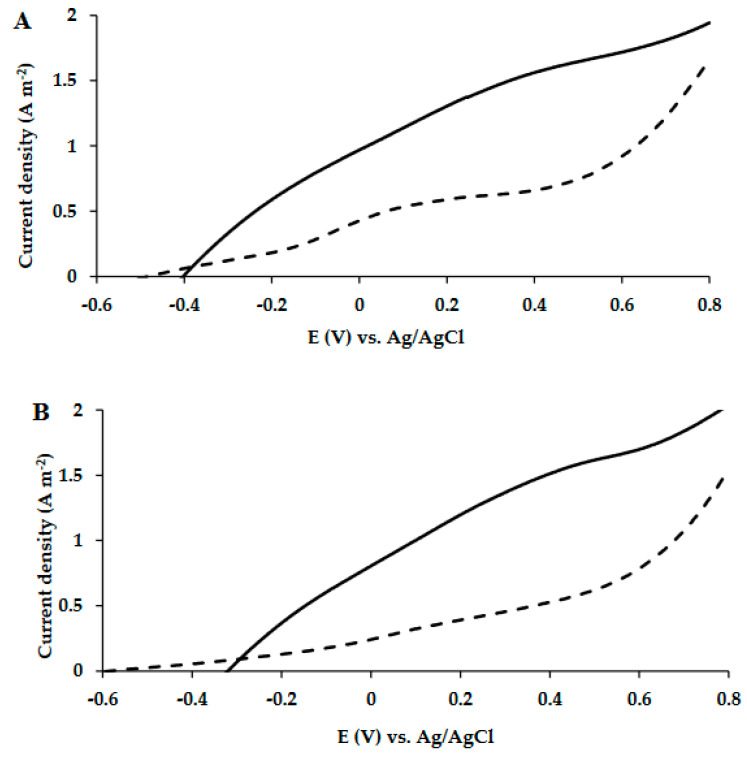
LSV polarization curves of MEC-SBP (**—**) and MEC control (**- - -**) supplied with artificial wastewater as the carbon source. LSV analysis was carried out on the 36th (**A**) and 57th (**B**) days of the MECs’ operation at a scan rate of 5 mV s^−1^ versus Ag/AgCl.

The MECs were fed with acetate as the carbon source for 32 days followed by artificial wastewater for another 46 days. In this second period, the artificial wastewater was replaced once a week and acetate was added twice a week. LSV measurements were performed at least once a week. LSV measurements on the 36th and 57th days of the MECs’ operation are shown in Figure 3A,B.

The results depicted in Figure 3A,B also showed that the MEC-SBP with the encapsulated anode led to higher currents than the MEC control. On the 36th and 57th days, under an applied voltage of 0.6 V vs. Ag/AgCl, the current densities of the MEC-SBP were 1.72 ± 0.15 and 1.70 ± 0.22 A m^−2^, respectively. In comparison, the MEC control yielded currents of only 0.92 ± 0.09 and 0.78 ± 0.01 A m^−2^, respectively. A slight decrease in the onset potential (from −0.04 to −0.2 V) of the MEC-SBP was seen when the devices were fed with acetate on the 14th and 27th days, respectively. The onset continued to decrease (to about −0.35 V) when the MECs were fed with wastewater. This phenomenon could explain the increase in the currents on the 36th and 57th days, compared to the 14th and 27th days.

Regarding the MEC control, the currents increased moderately from 0.3 A m^−2^ on the 14th day to 0.5 A m^−2^ on the 27th day. This may be attributed to the slow formation of the biofilm on the carbon-cloth anode, due to low bacterial inoculation, 0.6 mL (1.0 ± 0.05 OD) in a relatively high volume (100 mL) of the MEC control. In contrast, the MEC-SBP inoculation was introduced directly to the SBP capsule, which favored the biofilm formation because of the small void volume of the capsule (0.6 mL).

To summarize, on the 14th and 27th days, when the MECs were supplied with acetate, the MEC-SBP led to currents higher by 6.44-fold and 3.42-fold, respectively, compared to the MEC control. On the 36th and 57th days, when the MECs were supplied with artificial wastewater, the MEC-SBP led to currents higher by 1.87-fold and 2.18-fold, respectively, compared to the MEC control. These results showed that the MEC-SBP demonstrated better electrochemical performance than the MEC control.

In our previous studies, the bacterial anode was immobilized using natural polymers (alginate and chitosan) or encapsulated in a dialysis bag with different molecular-weight cutoffs. It was shown that when the bacteria on the carbon-cloth anode were immobilized using alginate and chitosan (1 mL alginate and chitosan was mixed with *G. sulfurreducens* (1 OD at 590 nm)), the current density at 0.6 V was 9.75 A m^−2^, whereas when the optical density was only 0.1 OD, the MEC produced a current density of only 5.04 A m^−2^ [26]. In the other study, the anode was made of carbon cloth combined with stainless steel and encapsulated in a dialysis bag with different molecular-weight cutoffs of 2 kDa, 14 kDa, and 50 kDa. In these MECs, the encapsulated anodes were inoculated with a suspension of *G. sulfurreducens* (10 mL with an optical density of 1 at 590 nm). The current densities obtained at 0.6 V vs. Ag/AgCl were 13.79 ± 0.30, 14.94 ± 0.49, and 16.34 ± 0.42 A m^−2^ for the anode encapsulated in a dialysis bag with molecular-weight cutoffs of 2 kDa, 14 kDa, and 50 kDa, respectively. In the MEC that employed a nonencapsulated anode, the current density was only 12.19 ± 0.49 A m^−2^ [27]. In the study of Zikmund et al. (2018), the MECs (28 mL) were operated under 0.9 V and were based on graphite-fiber brush anodes (2.5 cm length, 1.5 cm diameter, encased volume of 4.4 cm^3^) and carbon-felt anodes (7 cm^2^ cross-sectional surface area). The anodes were placed close to the cathode to reduce the electrolyte resistance between electrodes. The MEC based on the brush anode led to a current density of I_90_ = 4.2 ± 0.5 A m^−2^, compared to the felt anode, which led to I_90_ = 3.4 ± 0.1 A m^−2^ [34].

In summary, the inoculum concentration and favorable conditions for biofilm attachment are vital for the MEC electroactivity. As mentioned above, favorable conditions can be obtained by a high anode surface area [34], immobilization using alginate [26], inoculation in a dialysis bag [27], or inoculation into encapsulated SBP anodes, as described in our current study. These conditions also enabled earlier biofilm formation.

### 3.3. Reduction Currents and Hydrogen Production

**Figure 4:**
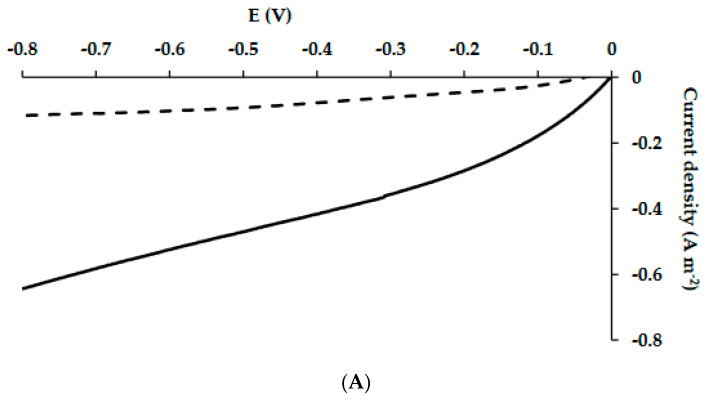

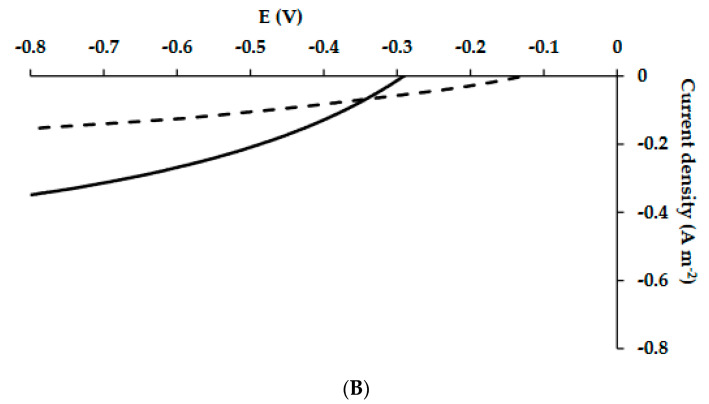
Reduction currents of MEC-SBP (**—**) and MEC-control (**- - -**) supplied with acetate as the sole carbon source (**A**) and artificial wastewater (**B**). The reduction currents were carried out at a scan rate of 5 mV s^−1^.

Reduction currents were measured at least once a week during the MECs’ operation, when the sole carbon source was acetate (Figure 4A), and when the MECs were fed with artificial wastewater (Figure 4B). The reduction current analyses were conducted when the MECs were in a configuration of a complete cell (two-electrode configuration).

The cathodic current of hydrogen evolution on the Pt electrode in a two-electrode configuration is shown in Figure 4A,B. At an applied maximum voltage difference of −0.8 V, the reduction currents to hydrogen gas in the MEC-SBP were higher than in the MEC control when cells were enriched with acetate, and to a lesser extent, also, when fed with artificial wastewater. The choice of −0.8 V represents a value at which the MEC had an advantage over conventional water-electrolysis cells (above 1.4 V). When the MECs were supplied with acetate, the reduction currents of the MEC-SBP were −0.64 ± 0.14 A m^−2^ (at −0.8 V), while the MEC control led to −0.12 ± 0.05 A m^−2^. When the MECs were fed with artificial wastewater, the MEC-SBP produced reduction currents of −0.35 ± 0.07 A m^−2^, 2.33-fold higher than the MEC control. The HERs (at −0.6 V) of the MECs, when supplied with acetate as the sole carbon or artificial wastewater, were calculated according to Equations (1) and (2) (given in the Materials and Methods section). In the MEC-SBP, the calculated HERs were 0.027 and 0.017 m^3^ m^−3^ day^−1^, respectively. In comparison, the MEC control led to only 0.006 and 0.005 m^3^ m^−3^ day^−1^, respectively.

**Figure 5:**
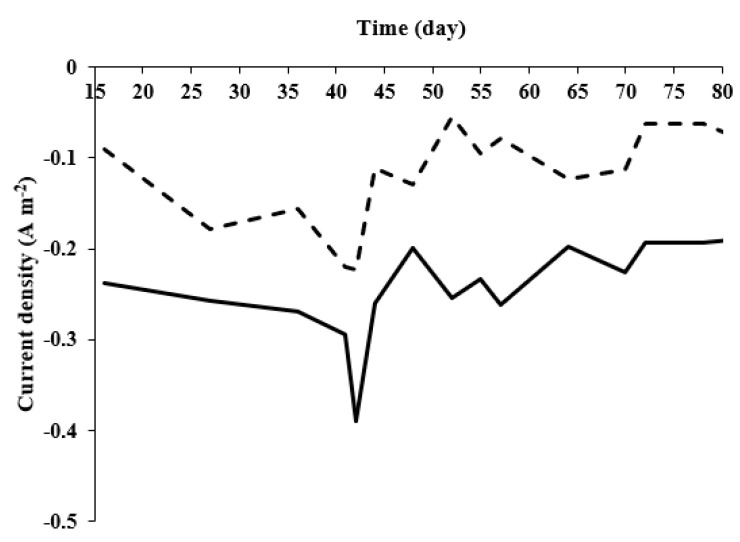
Reduction of protons to hydrogen currents under an applied potential of 0.6 V in MEC-SBP (**—**) and MEC control (**- - -**) during operation of 78 days.

Hydrogen production currents under an applied potential of 0.6 V were recorded from steady-state polarization curves measured at various times during the MEC-SBP and MEC control operation (Figure 5). The MECs were supplied with acetate as the carbon source for 32 days, followed by wastewater for another 46 days. The current changes seen in the graph are associated with the increase of the biofilm activity post-feeding steps. The current of the encapsulated anode in the MEC-SBP was higher (between 25–80%) than the bare carbon electrode in the MEC control. From these results, it is obvious that the encapsulation of bacterial anode in the MEC-SBP provides better long-term stability than the biofilm of the control anode.

Lim et al. (2022), constructed an MEC with two plain carbon-felt structures as anode and cathode (size 4.8 × 4.8 × 0.2 cm, projected area 25 cm^2^). The MEC working volume was 25 mL. The hydrogen evolution rate was 0.32 ± 0.01 m^3^ m^−3^ day^−1^ (6–11 days) and 0.37 ± 0.02 m^3^ m^−3^ day^−1^ (12–14 days) [13]. Yasri and Nakhla (2017) investigated an MEC employing granular activated carbon as a 3-dimensional (3D) anode. In different MECs, the anodes were doped with conductive calcium sulfide (CaS), iron sulfide (FeS), and magnetite (Fe_3_O_4_), and were compared to granular activated carbon without doping. In all anodes, the granular activated carbon (12 g) had a surface area of 900 m^2^ g^−1^ and a theoretical geometric surface area of the granular activated carbon per MEC anolyte chamber volume of 30.8 × 106 m^2^ m^−3^. The hydrogen production rate values were as follows, in decreasing order: 3-D CaS (0.54 ± 0.03 m^3^ m^−3^ d^−1^) > 3-D FeS (0.46 ± 0.02 m^3^ m^−3^ d^−1^) > 3-D Fe_3_O_4_ (0.36 ± 0.02 m^3^ m^−3^ d^−1^) > 3-D granular activated carbon (0.31 ± 0.01 m^3^ m^−3^ d^−1^) [35]. Zikmund et al. (2018) compared the bio-electroactivity of MECs based on a flat-felt anode with an MEC brush anode in a two-chamber, cubic type facility. The MECs with the brush anodes had a higher HER of 0.38 ± 0.02 m^3^ m^−3^ d^−1^, while the flat-felt anodes had only 0.32 ± 0.02 m^3^ m^−3^ d^−1^. They suggested that the main reason for the flat-felt anodes’ lower performance was substrate-limited mass transfer [34]. Wang et al. (2021) examined MEC performance with alkaline thermally pretreated sludge. The pretreatment was done at 90 °C and at 180 °C, allowing the release of more organic matter. The hydrogen yield using pretreated sludge at 90 °C was 0.44 m^3^ m^−3^ d^−1^, while at 180 °C it was only 0.31 m^3^ m^−3^ d^−1^ [36]. Gandu et al. (2020) examined HER performance of MECs based on immobilized anodes. The exoelectrogenic bacteria were immobilized using alginate and chitosan. This MEC led to a HER of 0.56 m^3^ m^−3^ d^−1^, while the MEC based on non-immobilized anodes led to 0.16 m^3^ m^−3^ d^−1^ [26]. Rozenfeld et al. (2021), studied MECs based on encapsulated anodes (carbon cloth combined with stainless steel) with a dialysis bag. An MFC based on dialysis bags with molecular weight cut-offs of 50 kDa led to a HER of 0.160 ± 0.009 m^3^ m^−2^ d^−1^, while the bare anode led to only 0.122 ± 0.004 m^3^ m^−2^ d^−1^ [27].

In conclusion, the HER of the MEC applying the SBP anode was higher than the MEC based on the control anode, which was not encapsulated. However, the HER of the MEC applying the SBP anode was 5 to10-fold less than the HER reported by other studies. It is important to note that the ratios of anode surface area to MEC working volume are important parameters for hydrogen evolution rates. We assume that expanding the carbon-cloth anode material surface area for biofilm attachment in the capsule and increasing the contact of the external titanium wire with the carbon cloth would improve the SBP-anode performance.

### 3.4. COD Removal

**Figure 6:**
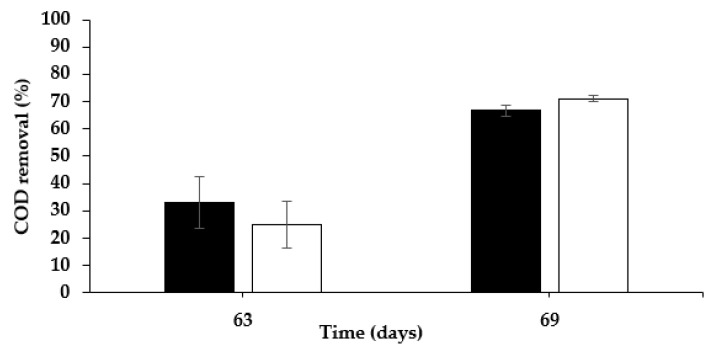
COD removal (%) in MEC-SBP (◼) and MEC-control (□) supplied with artificial wastewater. *p* value (*t* test): *p* > 0.05.

The COD inlet in the MEC-SBP and MEC control was 7400 ± 478 mg L^−1^. The COD was analyzed on the 59th, 63rd, and 69th days. COD removal on the 63rd day related to the COD on the 59th day, and the COD on the 69th day related to the COD on the 63rd day. All the samples were filtered, diluted according to the sample concentration, processed in the COD reactor, and analyzed for the absorbance intensity of the solution using spectrophotometry. As seen in Figure 6, the COD consumption on the 63rd day vs. the 59th day was 33 ± 9.5% in the MEC control, and 25 ± 8.5% in the MEC-SBP. On the 69th day vs. the 63rd day, it was 67 ± 2.0% and 71 ± 1.2%, respectively. There was no significance (*p* > 0.05) in the COD consumption in the MEC control vs. the MEC-SBP. The COD consumption rate for both was about the same, 650 ± 70 mg L^−1^.

Chaurasia and Mondal (2021) studied biohydrogen production using Ni, Ni-Co and Ni-Co-P electrodeposit cathodes in MFCs fed with sugar-industry wastewater. These MEC systems reportedly achieved ~47–50% COD removal. Initially, COD was 4850 ± 50 mg L^−1^, and after 7 days of operation was reduced to ~2425 mg L^−1^ [37]. Xie et al. (2021), showed that 34.26% of the COD provided was converted to electrical current in an 80-day period when the MEC was fed with rendering wastewater [38]. Keruthiga et al. (2021), investigated an MEC based on a modified carbon-cloth anode pasted with char and fed with wastewater. The reduction of COD was found to be correlated with acid concentration; increasing the acid concentration from 0.5 to 1.5% increased the COD reduction. The optimum acid concentration of 1.5% hydrolysed the organics effectively, which increased COD reduction to 76.8% [39]. Yu et al. (2021), studied a cylindrical-chamber MEC with a bed volume of 28 mL and a graphite brush anode of various sizes (surface area was 0.22 m^2^). COD removal efficiency in that case was 40.33% [40].

In conclusion, the COD removal in the MEC-SBP and the MEC control was similar to the COD removal reported in other studies.

### 3.5. Biofilm Viability on the Bacterial Anodes

**Figure 7:**
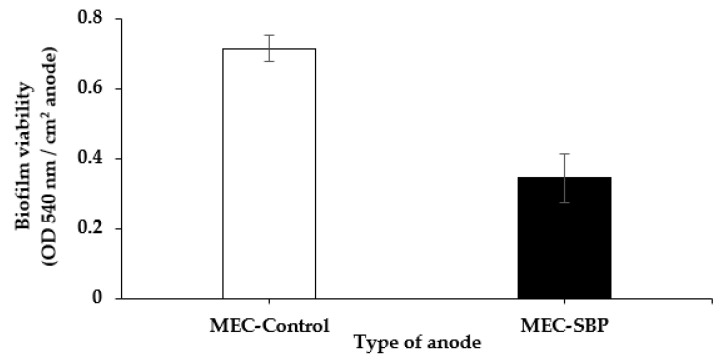
MTT analysis of the biofilm viability on the different anodes, MEC-SBP (◼) and MEC control (□) at the end of MEC operation. The results are normalized per one cm^2^ anode. *p*-value (*t* test): *p* < 0.05.

Biofilm viability was evaluated based on the reduction of tetrazolium salts by the bacterial hydrogenase. At the end of the experiment (day 78), the MEC-SBP capsule was cut, and the carbon cloth with the attached biofilm was removed. The bacterial anodes from the MEC control and MEC-SBP were gently washed in PBS (pH 6.8) to release the planktonic bacteria. The carbon-cloth anodes with their attached biofilms were transferred to MTT solution including tetrazolium salts. The bacterial hydrogenases reduced the yellowish crystals of the tetrazolium salts to purple, then the reduced tetrazolium salts were dissolved in a DMSO–ethanol solution. The absorbance intensity of the purple solution was examined using a spectrophotometer (Figure 7).

The results in Figure 7 show that the viability of the biofilm on the control anode was twice as high as that observed on the SBP anode, 0.71 ± 0.07 OD vs. 0.34 ± 0.04 OD, respectively. The lower bacterial viability on the MEC-SBP anode can be explained by the condensed carbon cloth at the bottom of the capsule. We assume that this pattern of folding inhibited bacterial attachment in the depth of the carbon cloth. However, the control carbon-cloth anode was bare and unfolded, with no limitation for bacterial attachment. In addition, the internal volume of the SBP was restricted to inoculation of only 0.6 mL (1 OD 590 nm).

Our previous study of a semi-single-chamber MEC was based on an anode encapsulated in a dialysis bag inoculated with *Geobacter sulfurreducens* (10 mL of 0.35 ± 0.05 OD), and presented a significant difference in the bacterial viability (using MTT analysis) between the anode types. When the MECs were fed with wastewater, the encapsulated anode’s viability was 2.5-fold higher than the nonencapsulated anode [27]. This finding might determine the biofilm-forming potential of the MEC-SBP anode once technical challenges are overcome.

### 3.6. Microbial Diversity on the Carbon-Cloth Anode

The MEC systems were inoculated with *Geobacter sulfurreducens* and fed with acetate for 32 days, followed by feeding with artificial wastewater for an additional 46 days. The artificial wastewater included *Staphylococcus aureus*, *Escherichia coli*, *Enterobacter cloacae*, and *Pseudomonas putida* to demonstrate wastewater flora.

**Figure 8:**
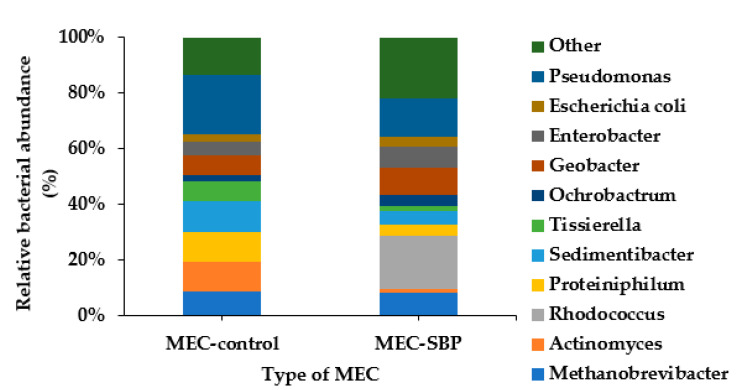
Relative bacterial abundance with respect to the genus level on biofilm anodes of the MEC control and MEC-SBP.

The microbial diversity on the MEC-SBP and MEC-control anodes was evaluated based on 16S rRNA at the end of the MECs’ operation (78 days). Operational taxonomic unit (OTU) readings were identified and phylogenetically classified. Five distinct phyla (*Proteobacteria*, *Firmicutes*, *Bacteroidetes*, *Actinobacteria*, and *Euryarchaeota*) were identified. The three most abundant phyla were *Proteobacteria, Firmicutes*, and *Actinobacteria*, with a relative distribution on the MEC-control anode of 32%, 32% and 14%, respectively. On the MEC-SBP anode, they were 40%, 15%, and 30%, respectively. Relative bacterial distribution with respect to the genus level of the anode biofilm is presented in Figure 8. Unidentified species or sequences with relative abundances of <2% were grouped as “Others/NA”.

*Geobacter* relates to the class *Deltaproteobacteria*; its distribution on the MEC control and MEC-SBP was 8% and 9%, respectively. The low percentage of *Geobacter* can explain the low reduction currents obtained, as seen in Figure 4. The currents obtained in the MEC control and MEC-SBP were −0.15 A m^−2^ and −0.35 A m^−2^ (at −0.8 V), respectively. However, *Rhodococcus erythropolis* was found in a relatively high distribution on the MEC-SBP (18%), while its distribution was negligible on the MEC control. We assume that the presence of *R*. *erythropolis* only on the MEC-SBP originated from self-contamination of the capsule. It is possible that *Rhodococcus erythropolis* also contributed to the currents found in the MEC-SBP. It was reported that *Rhodococcus erythropolis* was one of the dominant bacteria found on the biocathode of MFCs facilitating the mineralization of pentachlorophenol [41]. This bacterium was also identified in the microbial community of MFC biocathodes used for Cr(VI) reduction [42]. An additional species, *Rhodococcus pyridinivorans,* which was inoculated into MFCs, improved their power output. In this MFC, increasing the concentration of trehalose led to a 5.93-fold acceleration of the maximum power density, from 54.7 mW m^−2^ to 324.4 mW m^−2^ [43]. An interesting study by Taşkan and Taşkan showed that quorum quenching of the *Rhodococcus* sp. can control the biofilm thickness on the anode surface by inactivation of signal molecules among microorganisms, which reduces the production of extracellular polymeric substances. It was found that increases in *Rhodococcus* concentrations led to a reduction of the anode biofilm thickness and an abundance of dead bacteria. The best electrochemical activity (1924 mW m^−2^) was in an MFC with a biofilm thickness of 26 μm at 40 mg, using *Rhodococcus* immobilized in 10 mL sodium alginate [44].

In conclusion, we assume that the combination of *Geobacter* and *Rhodococcus* in the SBP anode led to a relatively higher current than in the control anode.

Concerning bacteria included in the artificial wastewater, *Staphylococcus* was found in negligible percentages, and *Escherichia* and *Enterobacter* were less than 10%. In contrast, the abundance of *Pseudomonas* was 22% on the bacterial anode of the MEC control, and only 13% on the MEC-SBP. *Pseudomonas* species are abundant in the microbial community of MFCs, and they are known to secrete electron mediators such as pyocyanin and phenazine with a redox potential of −0.03 V (versus SHE) [45,46,47]. *Pseudomonas alcaliphila* can excrete phenazine-1-carboxylic acid, which transfers electrons under alkaline conditions in the MFC. Results indicated that phenazine-1-carboxylic acid was a key factor for extracellular electron transfer [48].

### 3.7. SEM Analysis of the Bacterial Anodes

**Figure 9:**
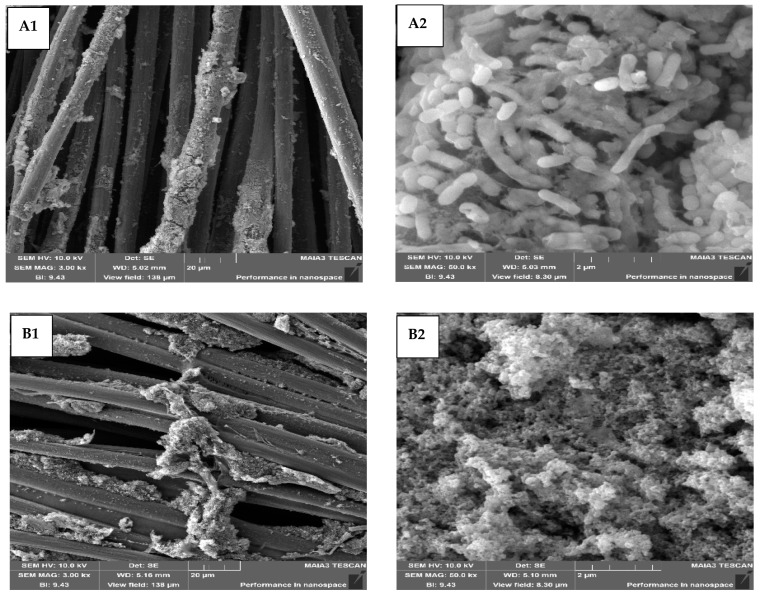
SEM images on the anode of the MEC control (**A1**,**A2**) and the MEC-SBP (**B1**,**B2**). Magnification: 3 kx (**A1**,**B1**) and 50 kx (**A2**,**B2**).

At the end of the MECs’ operation (78 days), the anodes were dehydrated and prepared for visualization by SEM. The images are shown in Figure 9. The SEM images of the MEC control (Figure 9(A1,A2); magnification 3 kx and 50 kx, respectively) showed bacterial aggregation, especially on the carbon fibers (A1); and bacterial cells with relatively low matrices (A2). However, the SEM images of the SBP anode showed a massive biofilm on and between the fibers (Figure 9(B1)), and a very dense biofilm (Figure 9(B2)). It is important to note that there was still space for substrate access despite the massive biofilm on the SBP anode. Moreover, the biofilm’s morphological structure was quite different in the two systems. MTT analysis showed higher biofilm viability (2-fold) on the control anode compared to the biofilm on the SBP anode. In contrast, SEM images showed less biofilm on the control anode, indicating different biofilm development over time. We assume that the biofilm on the control anode was looser, probably influencing electron flux to the carbon-cloth anode material.

Ishii et al. revealed that the biofilm on the anode was nonhomogeneous at the beginning of MEC operation (11 days). There were many large aggregates, and the electrode was partially covered by the bacterial cells. After long-term operation (216 days), there was an increase in the coverage area of *G. sulfurreducens* cells, resulting in a dense biofilm on the anode. They indicated that the limiting current density changed proportionally to biomass densities on the anode [49]. Liu et al. showed bacterial anode images on different electrode materials, graphite rods, and carbon-fiber veils. On the carbon rod, a thick and dense biofilm was observed, but on the carbon-fiber veil, the biofilm colonized every carbon fiber with a thickness of more than 10 µm. There was more space between intersectional carbon-fiber biofilm on the carbon fibers. This porous structure may provide better substrate access, resulting in high current density [50]. Chang et al. modified a carbon-cloth anode surface by screen-printing reduced graphene oxide, and calcination using an atmospheric-pressure plasma jet. Both treatments significantly increased the hydrophilicity and surface area of the effective materials for bacterial adhesion [51].

## 4. Conclusions

In this study, a novel bacterial anode encapsulation technology based on a small bioreactor platform (SBP) was developed. To the best of our knowledge, and after a survey of the literature, this approach has not been reported before. The capsule membrane physically separates the microbial culture inside the capsule, including the carbon-cloth anode material from the natural microorganisms in the wastewater, while enabling nutrient diffusion.

The MECs based on the SBP anode, as well as the MECs based on the nonencapsulated anode (MEC control), were fed with *Geobacter* medium supplied with acetate for 32 days, and then with artificial wastewater for another 46 days.

LSV measurements of the MEC-SBP fed with artificial wastewater produced 1.70 ± 0.22 A m^−2^ (at 0.6 V), i.e., double the rate of the control. The HERs of the MEC-SBP, when supplied with acetate as the sole carbon or with artificial wastewater, were 0.027 and 0.017 m^3^ m^−3^ day^−1^, respectively. In comparison, the MEC control led to only 0.006 and 0.005 m^3^ m^−3^ day^−1^, respectively. The COD consumption rate for both MECs was about the same at 650 ± 70 mg L^−1^. Biofilm viability on the control anode was twice as high as that observed on the SBP anode. The microbial diversity on the MEC-SBP and MEC-control anodes showed that the relative distribution of *Geobacter* was only 10%, which can explain the relatively low currents.

To increase the currents in the MEC-SBP, SBP-anode technology must overcome several obstacles, such as expanding the carbon-cloth anode material surface area for biofilm attachment, a proper method for sterilization of the capsule, and increasing the contact of the external titanium wire with the carbon cloth.

The SBP-anode approach may protect the exoelectrogenic bacterial anode from the invasion of non-exoelectrogenic bacteria that may reduce the electron transfer from the bacteria to the carbon-cloth anode. The non-exoelectrogenic bacteria can also compete with the exoelectrogenic bacterial anode for nutrients. In addition, the SBP anode is stable in withstanding the shear and friction forces found in domestic wastewater treatment plants. All these improvements may accelerate electron transfer and the HER.

## 5. Patents

1. PCT IL2010/256 (W.O./2010/122545).
2. PCT/IL2021/050911.

## Data Availability

Data is contained within the article.

## References

[1] Guo K., Prévoteau A., Rabaey K. (2017). A novel tubular microbial electrolysis cell for high rate hydrogen production. J. Power Sources.

[2] Wu Z., Wang S., Zhao J., Chen L., Meng H. (2014). Synergistic effect on thermal behavior during co-pyrolysis of lignocellulosic biomass model components blend with bituminous coal. Bioresour. Technol..

[3] Logan B.E. (2008). Microbial Fuel Cells.

[4] Al-Shara N.K., Sher F., Yaqoob A., Chen G.Z. (2019). Electrochemical investigation of novel reference electrode Ni/Ni(OH)₂ in comparison with silver and platinum inert quasi-reference electrodes for electrolysis in eutectic molten hydroxide. Int. J. Hydrogen Energy.

[5] Sher F., Al-Shara N.K., Iqbal S.Z., Jahan Z., Chen G.Z. (2020). Enhancing hydrogen production from steam electrolysis in molten hydroxides via selection of non-precious metal electrodes. Int. J. Hydrogen Energy.

[6] Liu H., Grot S., Logan B.E. (2005). Electrochemically assisted microbial production of hydrogen from acetate. Environ. Sci. Technol..

[7] Rivera I., Bakonyi P., Cuautle-Marín M.A., Buitrón G. (2017). Evaluation of various cheese whey treatment scenarios in single-chamber microbial electrolysis cells for improved biohydrogen production. Chemosphere.

[8] Santoro C., Arbizzani C., Erable B., Ieropoulos I. (2017). Microbial fuel cells: From fundamentals to applications. A review. J. Power Sources.

[9] Friman H., Schechter A., Ioffe Y., Nitzan Y., Cahan R. (2013). Current production in a microbial fuel cell using a pure culture of *Cupriavidus basilensis* growing in acetate or phenol as a carbon source. Microb. Biotechnol..

[10] Bhatia S.K., Jagtap S.S., Bedekar A.A., Bhatia R.K., Rajendran K., Pugazhendhi A., Rao C.V., Atabani A.E., Kumar G., Yang Y.H. (2021). Renewable biohydrogen production from lignocellulosic biomass using fermentation and integration of systems with other energy generation technologies. Sci. Total Environ..

[11] Fonseca E.U., Yang W., Wang X., Rossi R., Logan B.E. (2021). Comparison of different chemical treatments of brush and flat carbon electrodes to improve performance of microbial fuel cells. Bioresour. Technol..

[12] Hoang A.T., Nižetić S., Ng K.H., Papadopoulos A.M., Le A.T., Kumar S., Hadiyanto H., Pham V.V. (2022). Microbial fuel cells for bioelectricity production from waste as sustainable prospect of future energy sector. Chemosphere.

[13] Lim S.S., Fontmorin J.-M., Salehmin M.N.I., Feng Y., Scott K., Yu E.H. (2022). Enhancing hydrogen production through anode fed-batch mode and controlled cell voltage in a microbial electrolysis cell fully catalysed by microorganisms. Chemosphere.

[14] Lim S.S., Fontmorin J.-M., Izadi P., Daud W.R.W., Scott K., Yu E.H. (2020). Impact of applied cell voltage on the performance of a microbial electrolysis cell fully catalysed by microorganisms. Int. J. Hydrogen Energy.

[15] Wang W., Lee D.J., Lei Z. (2022). Integrating anaerobic digestion with microbial electrolysis cell for performance enhancement: A review. Bioresour. Technol..

[16] Wang H.C., Cui D., Han J.L., Cheng H.Y., Liu W.Z., Peng Y.Z., Chen Z.B., Wang A.J. (2019). A_2_O-MBR as an efficient and profitable unconventional water treatment and reuse technology: A practical study in a green building residential community. Resour. Conserv. Recycl..

[17] Schechter M., Schechter A., Rozenfeld S., Efrat E., Cahan R. (2014). Anode Biofilm. Technology and Application of Microbial Fuel Cells.

[18] Menashe O., Kurzbaum E. (2014). Small-bioreactor platform technology as a municipal wastewater additive treatment. Water Sci. Technol..

[19] Menashe O., Rosen-Kligvasser J., Kurzbaum E., Suckeveriene R.Y. (2021). Structural properties of a biotechnological capsule confined by a 3D-cellulose acetate membrane. Polym. Adv. Technol..

[20] Wang A., Liu W., Ren N., Cheng H., Lee D.-J. (2010). Reduced internal resistance of microbial electrolysis cell (MEC) as factors of configuration and stuffing with granular activated carbon. Int. J. Hydrogen Energy.

[21] Cai H., Wang J., Bu Y., Zhong Q. (2013). Treatment of carbon cloth anodes for improving power generation in a dual-chamber microbial fuel cell. J. Chem. Technol. Biotechnol..

[22] Guan Y.F., Zhang F., Huang B.C., Yu H.Q. (2019). Enhancing electricity generation of microbial fuel cell for wastewater treatment using nitrogen-doped carbon dots-supported carbon paper anode. J. Clean. Prod..

[23] Baek G., Saikaly P.E., Logan B.E. (2021). Addition of a carbon fiber brush improves anaerobic digestion compared to external voltage application. Water Res..

[24] Scott K., Rimbu G.A., Katuri K.P., Prasad K.K., Head I.M. (2007). Application of modified carbon anodes in microbial fuel cells. Process Saf. Environ. Prot..

[25] Rozenfeld S., Ouaknin Hirsch L., Gandu B., Farber R., Schechter A., Cahan R. (2019). Improvement of microbial electrolysis cell activity by using anode based on combined plasma-pretreated carbon cloth and stainless steel. Energies.

[26] Gandu B., Rozenfeld S., Ouaknin Hirsch L., Schechter A., Cahan R. (2020). Immobilization of bacterial cells on carbon-cloth anode using alginate for hydrogen generation in a microbial electrolysis cell. J. Power Sources.

[27] Rozenfeld S., Gandu B., Hirsch L.O., Dubrovin I., Schechter A., Cahan R. (2021). Hydrogen production in a semi-single-chamber microbial electrolysis cell based on anode encapsulated in a dialysis bag. Int. J. Energy Res..

[28] Menashe O., Kurzbaum E. (2016). A novel bioaugmentation treatment approach using a confined microbial environment: A case study in a Membrane Bioreactor wastewater treatment plant. Environ. Technol..

[29] Rozenfeld S., Teller H., Schechter M., Farber R., Krichevski O., Schechter A., Cahan R. (2018). Exfoliated molybdenum di-sulfide (MoS_2_) electrode for hydrogen production in microbial electrolysis cell. Bioelectrochemistry.

[30] Pasupuleti S.B., Srikanth S., Venkata Mohan S., Pant D. (2015). Development of exoelectrogenic bioanode and study on feasibility of hydrogen production using abiotic VITO-CoRE^TM^ and VITO-CASE^TM^ electrodes in a single chamber microbial electrolysis cell (MEC) at low current densities. Bioresour. Technol..

[31] Zhao Y., Dong Z., Wang Y., Li J., An X., Yang D. (2019). Process kinetics for the electrocatalytic hydrogen evolution reaction on carbon-based Ni/NiO nanocomposite in a single-chamber microbial electrolysis cell. Int. J. Hydrogen Energy.

[32] Farber R., Dabush-Busheri I., Chaniel G., Rozenfeld S., Bormashenko E., Multanen V., Cahan R. (2019). Biofilm grown on wood waste pretreated with cold low-pressure nitrogen plasma: Utilization for toluene remediation. Int. Biodeterior. Biodegrad..

[33] Katz H., Farber R., Chaniel G., Ankar Y., Cohen H., Cahan R. (2018). Rhamnolipid-enhanced *Pseudomonas putida* biofilm formation on hydrophilic surfaces with toluene as the bacterium’s sole carbon source. Int. Biodeterior. Biodegrad..

[34] Zikmund E., Kim K.Y., Logan B.E. (2018). Hydrogen production rates with closely-spaced felt anodes and cathodes compared to brush anodes in two-chamber microbial electrolysis cells. Int. J. Hydrogen Energy.

[35] Yasri N.G., Nakhla G. (2017). The performance of 3-D graphite doped anodes in microbial electrolysis cells. J. Power Sources.

[36] Wang H., Liu J., Zhang Z., Li J., Zhang H., Zhan Y. (2021). Alkaline thermal pretreatment of waste activated sludge for enhanced hydrogen production in microbial electrolysis cells. J. Environ. Manag..

[37] Chaurasia A.K., Mondal P. (2021). Hydrogen gas production from paper–pulp industry wastewater by electrodeposited cathodes in MECs. Lect. Notes Mech. Eng..

[38] Xie A., Deaver J.A., Miller E., Popat S.C. (2021). Evaluation of electrical current production in microbial electrolysis cells fed with animal rendering wastewater. Chemosphere.

[39] Keruthiga K., Mohamed S.N., Gandhi N.N., Muthukumar K. (2021). Sugar industry waste-derived anode for enhanced biohydrogen production from rice mill wastewater using artificial photo-assisted microbial electrolysis cell. Int. J. Hydrogen Energy.

[40] Yu Z., Liu W., Shi Y., Wang B., Huang C., Liu C., Wang A. (2021). Microbial electrolysis enhanced bioconversion of waste sludge lysate for hydrogen production compared with anaerobic digestion. Sci. Total Environ..

[41] Huang L., Chai X., Quan X., Logan B.E., Chen G. (2012). Reductive dechlorination and mineralization of pentachlorophenol in biocathode microbial fuel cells. Bioresour. Technol..

[42] Romo D.M., Gutiérrez N.H., Pazos J.O., Figueroa L.V., Ordóñez L.A. (2019). Bacterial diversity in the Cr(VI) reducing biocathode of a Microbial Fuel Cell with salt bridge. Rev. Argent. Microbiol..

[43] Cheng P., Shan R., Yuan H.R., Deng L.F., Chen Y. (2018). Enhanced *Rhodococcus pyridinivorans* HR-1 anode performance by adding trehalose lipid in microbial fuel cell. Bioresour. Technol..

[44] Taşkan B., Taşkan E. (2021). Inhibition of AHL-mediated quorum sensing to control biofilm thickness in microbial fuel cell by using *Rhodococcus sp*. BH4. Chemosphere.

[45] Rabaey K., Boon N., Siciliano S.D., Verhaege M., Verstraete W. (2004). Biofuel cells select for microbial consortia that self-mediate electron transfer. Appl. Environ. Microbiol..

[46] Semenec L., Franks A.E. (2015). Delving through electrogenic biofilms: From anodes to cathodes to microbes. Aims Bioeng..

[47] Torres C.I., Krajmalnik-Brown R., Parameswaran P., Marcus A.K., Wanger G., Gorby Y.A., Rittmann B.E. (2009). Selecting anode-respiring bacteria based on anode potential: Phylogenetic, electrochemical, and microscopic characterization. Environ. Sci. Technol..

[48] Zhang T., Zhang L., Su W., Gao P., Li D., He X., Zhang Y. (2011). The direct electrocatalysis of phenazine-1-carboxylic acid excreted by *Pseudomonas alcaliphila* under alkaline condition in microbial fuel cells. Bioresour. Technol..

[49] Ishii S., Watanabe K., Yabuki S., Logan B.E., Sekiguchi Y. (2008). Comparison of electrode reduction activities of *Geobacter sulfurreducens* and an enriched consortium in an air-cathode microbial fuel cell. Appl. Environ. Microbiol..

[50] Liu Y., Harnisch F., Fricke K., Schröder U., Climent V., Feliu J.M. (2010). The study of electrochemically active microbial biofilms on different carbon-based anode materials in microbial fuel cells. Biosens. Bioelectron..

[51] Chang S.H., Huang B.Y., Wan T.H., Chen J.Z., Chen B.Y. (2017). Surface modification of carbon cloth anodes for microbial fuel cells using atmospheric-pressure plasma jet processed reduced graphene oxides. RSC Adv..

